# Avoiding health technology assessment: a global survey of reasons for not using health technology assessment in decision making

**DOI:** 10.1186/s12962-021-00308-1

**Published:** 2021-09-22

**Authors:** Yot Teerawattananon, Chris Painter, Saudamini Dabak, Trygve Ottersen, Unni Gopinathan, Lumbwe Chola, Kalipso Chalkidou, Anthony J. Culyer

**Affiliations:** 1 Health Intervention and Technology Assessment Program (HITAP), Ministry of Public Health, Nonthaburi, Thailand; 2 Saw Swee Hock School of Public Health, National University of Singapore, Singapore, Singapore; 3 Norwegian Institute of Public Health, Oslo, Norway; 4 The Global Fund to Fight AIDS, Tuberculosis and Malaria, Geneva, Switzerland; 5 Imperial College London, London, UK; 6 University of York, York, UK

## Abstract

**Introduction:**

Despite the documented benefits of using health technology assessments (HTA) to inform resource allocation in health care systems, HTA remains underused, especially in low- and middle-income countries. A survey of global health practitioners was conducted to reveal the top reasons (“excuses”) that they had heard from colleagues, policymakers or other stakeholders for not using HTA in their settings.

**Methods:**

There were 193 respondents to the survey. Most responses were from individuals in research organisations (37%), ministries of health (27%) and other government agencies (14%). Participants came from Southeast Asia (40%), the Western Pacific (30%), Africa (15%), Europe (7%), the Americas (7%) and the Eastern Mediterranean region (2%).

**Results:**

The top five reasons encountered by respondents related to lack of data, lack of technical skills for HTA, the technocratic nature of the work, the lack of explicit decision rules and the perception that HTA puts a “price on life”.

**Conclusions:**

This study aimed to understand and address the top reasons for not using HTA. They fall into three categories: (1) misconceptions about HTA; (2) feasibility issues; and (3) values, attitudes and politics. Previous literature has shown that these reasons can be addressed when identified, and even imperfect HTA analyses can provide useful information to a decision-maker.

**Supplementary Information:**

The online version contains supplementary material available at 10.1186/s12962-021-00308-1.

## Introduction

Health technology assessment (HTA) is a systematic approach for evaluating the properties, benefits and costs of health technologies and services. It is a multidisciplinary process that incorporates scientific, social, economic and ethical considerations in order to inform policy making decisions about whether particular interventions represent good value for money. HTA can help health systems to use their scarce resources more efficiently, in turn helping to maximise population health outcomes within a budget constraint. HTA can make healthcare decisions more transparent and defensible, and can also be used in price negotiations with manufacturers. The benefits of HTA have been demonstrated in low-, middle- and high-income countries [1–5]. A key requirement for the institutionalisation of HTA within country healthcare systems is that it requires (a) access to a non-trivial level of institutional capacity, including skilled researchers with the prerequisite training in key topics (such as health economics and evidence synthesis) and (b) governance structures and legislative measures to support these efforts [6].

A 2015 World Health Organization (WHO) survey found that approximately 80% of responding countries had a formal process for collecting evidence on new health technologies and services in their country. Few, however, referred to this process as HTA and the types of evidence included in these processes varied considerably, with most focussing primarily on the safety and clinical effectiveness of interventions [7]. HTA, by contrast, is a multidisciplinary process using explicit methods to determine the value of a health technology in terms of its potential impact on people’s health. The goals of HTA are to inform decision-making and promote an equitable, efficient, and high-quality health system [8]. An economic evaluation component is typically included in the processes of some of the world’s best known and methodologically comprehensive HTA agencies (who also have strong political mandates), such as the National Institute for Health and Care Excellence (NICE) and the Canadian Agency for Drugs and Technologies in Health (CADTH). The purpose of the economic element is not to assign monetary values to inputs and outcomes for their own sake but to enable comparisons between options and, in particular, to reduce the risk of adopting interventions whose resource requirements prohibit the adoption of other interventions with better or fairer health outcomes.

Despite the documented benefits that using HTA can have, [9–11] barriers exist in achieving political buy-in. One of the arguments used against HTA is that the methods, for economic evaluations in particular, can be complex and difficult to communicate in simple terms—and policymakers may be wary of using information of this type to inform policy decisions that they cannot easily relay to the public. In other countries, such as the USA, institutions that would make use of HTA evidence and cost-effectiveness thresholds have been effectively labelled as ‘death panels’ with other similarly unappealing tags, [12] creating significant political opposition to the use of HTA in policymaking. Arguments of this sort against HTA and economic evaluation in general vary substantially between countries, and depend on local political culture and values [13]. Tackling entrenched ideological opposition is not a task for which HTA practitioners are well-equipped, other than by supplying practical evidence for its usefulness, so a broadly supportive constitutional and political environment is necessary for its effective adoption.

Over the course of recent decades, the use of HTA has afforded countries at varying levels of income the ability to provide healthcare to their citizens at reasonable cost. We therefore wanted to learn why HTA remains underused in other countries. A team at the Norwegian Institute of Public Health (NIPH) has initiated efforts to improve the understanding of these arguments, or ‘top excuses’, for not using HTA. The team, together with a broad range of international collaborators, has so far sought to identify a shortlist of these top reasons and, using global and local HTA experts to describe them in a narrative format. Formal HTA processes have been introduced in several low- and middle-income countries (LMICs) in recent years, [4] and the aim of this research was to complement the efforts of the NIPH by encompassing a wide scope of perspectives, particularly those from LMICs, using an online survey, and providing a quantitative analysis of responses. LMICs may benefit even more from the use of HTA methods than high-income countries, given a need to gain maximum leverage in terms of health from very limited resources. Understanding the perceived barriers to the use of HTA in LMICs therefore becomes a priority. To this end, we conducted a survey of global health practitioners to learn what the most common reasons were, as perceived by respondents, for not using HTA methods (particularly cost-effectiveness analysis) that they had encountered in their settings. Improving the understanding of these reasons may allow these concerns to be addressed or allayed in a systematic manner.

## Methods

### Survey design

A questionnaire was designed to learn about health policy practitioner perspectives on the top reasons or ‘excuses’, as perceived by the respondents, they encountered for not using HTA and cost-effectiveness analysis (Additional file 1). Participants at the 2020 Prince Mahidol Award Conference (PMAC) were asked four questions. PMAC is an annual international conference focussed on policy-related health issues, for a target audience of policymakers, senior officers, and staff of national bodies who are responsible for the decisions of resource allocation in ministries of finance, health and other relevant agencies (such as HTA bodies, universities and industry). Questions asked respondents to select the most common “excuses” (or reasons, hereinafter) for not using HTA that they had encountered in their setting, from a predefined list of reasons (Additional file 2). They were then asked to choose which of the listed reasons they personally rejected most strongly. The final two questions were free-form questions that asked the respondents to explain why they rejected the selected reason, and whether there were any reasons they had encountered for not using HTA that were not already listed in this survey. The original list of reasons was drawn up by the team at NIPH, through its analysis on the topic with leading global experts, as indicated above. After the initial piloting, the second stage involved adapting the original list for the purpose of the online survey. In addition to these questions, survey participants were asked to provide demographic information to enable analysis of sub-groups.

### Target group

The target group for the survey was practitioners in the field of HTA from around the world.

### Sample and survey administration

The survey was first fielded in English, in an online format to attendees of the 2020 PMAC held in Bangkok, Thailand on 28 January–2 February. The duration of the survey was January through August 2020. The survey was disseminated online via a website with a video explaining the purpose of the survey (link: https://www.goforcea.club/). The video and website were deliberately made simple and attractive to maximise responses. The time estimated for respondents to complete the survey was estimated to be less than 10 min. The survey was disseminated to PMAC 2020 attendees and collaborating research institutions across the world via email and promoted on the PMAC 2020 and Health Intervention and Technology Assessment Program (HITAP) websites and social media platforms (Twitter and Facebook). Reminder emails were sent to invitees when the survey response period was extended. To incentivise participation in the survey, a prize to attend an HTA training in Thailand was offered and two winners were selected through a lucky draw of the total number of respondents.

### Ethics approval and disclosures

Ethics approval was not sought as the questions were general and not harmful to respondents. A data disclosure statement was included on the webpage informing survey participants that their responses will be reported anonymously, for non-commercial purposes in aggregate form. Patients or the public were not involved in the design, or conduct, or reporting, or dissemination plans of our research.

### Data analysis

Descriptive statistics, content analysis of open-ended questions, sub-group analyses and the cross-tabulation of responses were extracted from the data. Sub-group analyses were conducted of responses to questions according to the income level of the respondents’ workplace countries and the nature of their workplaces. The study was not powered to detect statistically significant differences between sub-groups and no statistical tests were performed. For the responses to open-ended questions, content analysis was performed by two reviewers to classify the responses into thematic areas, which were aggregated into three broad categories for further interpretation: (1) misconceptions about HTA; (2) feasibility issues; and (3) values, attitudes and politics. These three categories were constructed after an initial review of the responses to the survey. Any conflicts from the review were resolved through discussion to reach agreement. An individual’s response could be allocated into multiple categories as they could discuss several themes in their free-form responses. Analysis was conducted using Microsoft Excel and the geographic distribution of participants was developed using Tableau.

## Results

### Respondent characteristics

**Fig. 1:**
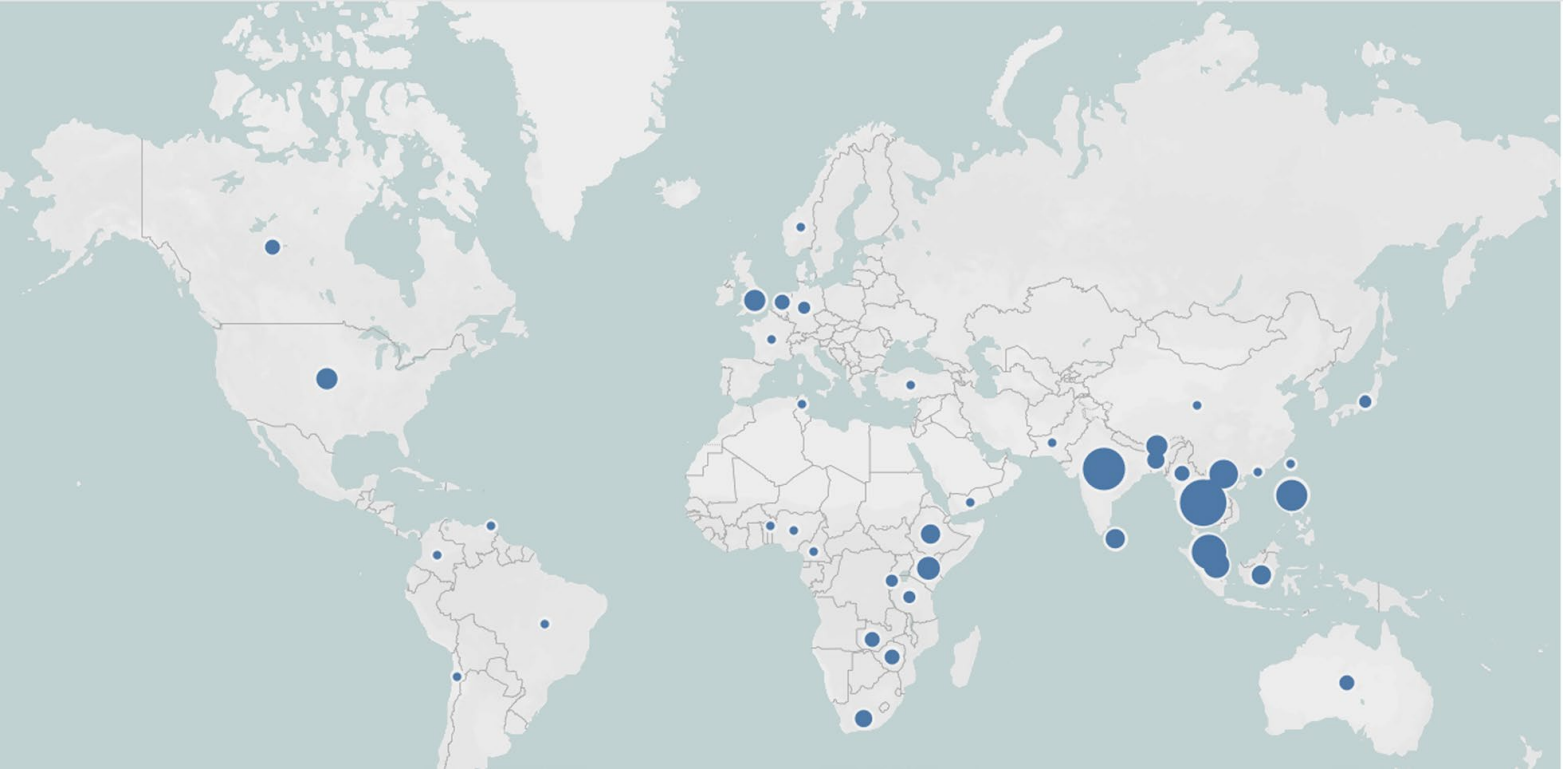
Geographical distribution of survey respondents by country

There were 193 respondents from 42 countries, most from research organisations (37%), ministries of health (27%) and other government agencies (14%). In terms of geographical representation (grouped using WHO’s classification of countries by region), most participants came from Southeast Asia (40%), followed by the Western Pacific (30%), Africa (15%), Europe (7%), the Americas (7%) and the Eastern Mediterranean region (2%). Most respondents came from countries classified by the World Bank as lower middle-income countries (45%) and upper middle-income countries (31%), with a fifth coming from high income countries and 4% from low-income countries. The geographical distribution of the survey respondents is displayed in Fig. 1.

**Fig. 2:**
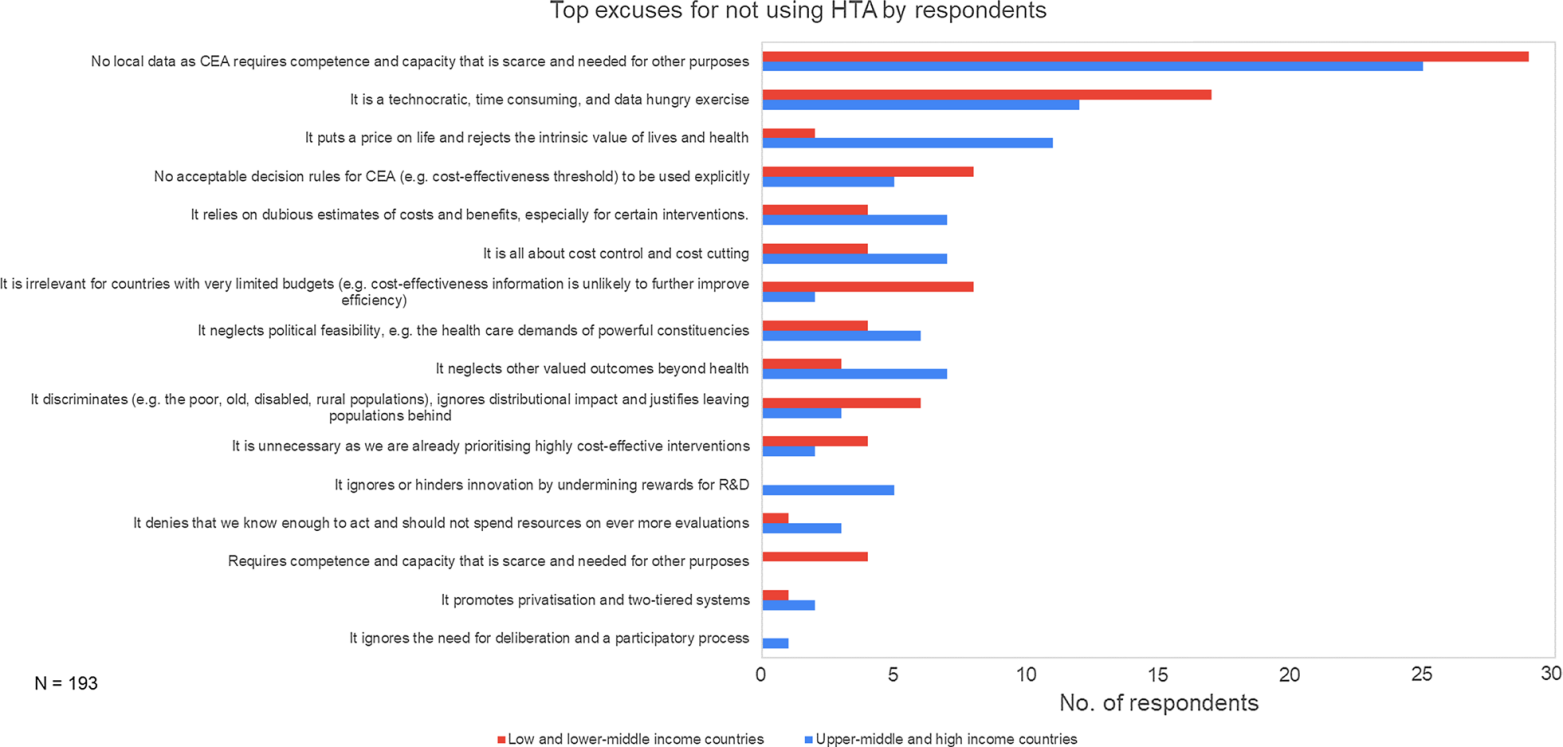
Survey respondents’ top reasons encountered for not using HTA in their setting

The most frequently cited reasons for not using HTA related to lack of data and technical skills for HTA, the technocratic nature of the work, the perception that HTA puts a “price on life” and the lack of acceptable decision rules, as shown in Fig. 2. Other common reasons included the objection that HTA is “all about cost control and cost cutting” and that it relies on dubious estimates of costs and benefits. Compared to upper middle- and high-income country respondents, respondents from lower middle- and low-income countries more frequently cited data limitations (31% as opposed to 26%) and the technocratic and resource-intensive nature of HTA (18% and 12%, respectively) as reasons for not using HTA. Upper middle- and high-income country respondents more frequently encountered arguments against HTA based on it being merely a tool for cost control, its lack of focus on other health outcomes and again that it put a “price on life”.

**Fig. 3:**
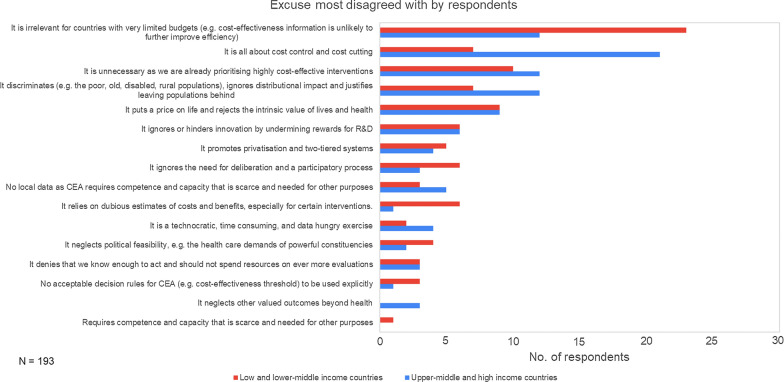
Top reasons for not using HTA in their setting most strongly rejected

However, the reasons that respondents rejected most strongly (Fig. 3) included the claim that HTA is not relevant in countries with limited budget (18%) and that HTA is only about costs control and cost-cutting (15%), as shown in Fig. 3. In offering their views on why they rejected these reasons, the largest group of respondents, close to a third (28%), contended that there is limited understanding of HTA in the settings where they operate and argue that HTA does not only relate to efficiency but encompasses other health concerns such as equity and feasibility of implementation. Respondents also highlighted the importance of evidence in healthcare decision-making, maximising the potential of limited budgets and unlocking efficiencies. Researchers were most likely to say that they rejected the top five reasons most strongly identified due to, what they felt, was a limited understanding of HTA. Respondents from ministries of health or other government agencies, on the other hand, were most likely to contend that HTA consisted of more than just economic considerations in their rebuttal of the top five HTA reasons that they rejected most strongly.

When asked for additional reasons, not covered in the list provided, most respondents stated that the list was comprehensive (66%). About 16% of the respondents, however, felt that HTA research would be or is ignored by policymakers in their settings, with others emphasising the limitations placed by the lack of reliable data (8%) and that HTA was complicated (5%).

## Discussion

This study aimed to understand and address the top reasons for not using HTA, as perceived by health policy specialists. The top reasons can largely be split into three categories of issues: (1) misconceptions about HTA; (2) feasibility issues; and (3) values, attitudes and politics. Low and lower-middle income country respondents cited top reasons in category 2 more frequently than upper-middle and high-income country respondents (65% of responses, compared to 50%). Upper-middle and high-income country respondents cited reasons in category 1 and category 3 more frequently than low and lower-middle income country respondents (28% compared to 23%, and 22% compared to 12%, respectively). These differences may not be statistically significant.

Category 1 consists of commonly held misconceptions about HTA: that it’s primarily concerned with cost control and cost-cutting, that it is not relevant for countries with limited budgets or that it pays insufficient attention to equity. These reasons poorly reflect the country-level experience of HTA, which actually point to the opposite. It is possible for healthcare costs to rise as a result of HTA and economic evaluations. A cost-effectiveness or willingness-to-pay threshold (routinely used in HTA analyses) is an explicit expression of the decision maker’s required health benefits from an intervention to justify increased spending. With respect to relevance for countries with limited budgets, such countries potentially have the most to gain from the use of HTA, as the health opportunity cost from misallocating resources in these countries is much higher than in high income countries which can afford to offer many more health technologies. It is never too early for countries to start using HTA; indeed, as expressed in what is referred to as “Buxton’s Law”, “it is always too early to assess a new technology, until suddenly it’s too late!” [14].

Furthermore, HTA is not restricted to maximising total health benefits. Indeed, equity issues have shaped many decisions including those on life-saving interventions such as renal replacement therapy in Thailand, or to support patients with rare diseases [15]. In countries like Thailand, equity issues are addressed through criteria for selecting topics for HTA assessment, and the issues receive further attention through stakeholder engagement which is a staple of HTA research in the country [16]. Evidence generated from HTA has informed the decisions even when the interventions have not been cost-effective. In recent years there have been methodological advances towards addressing equity issues quantitatively (e.g., distributional and extended cost-effectiveness analyses) [17–19].

Category 2 includes resource, data and time constraints—capacity building activities, academic research and the production of public goods can all assist in overcoming these barriers to HTA adoption. The lack of data and local technical capacity for conducting HTA, which was the most cited issue in the survey results among LMICs, remains a challenge [20]. However, over the past decade there have been strides made in advancing technical capacity for HTA among LMICs. Trainings have been conducted and long-term HTA courses have created a pool of researchers that can contribute to HTA in countries [3, 21–23]. On-the-job training has been catalytical for many countries in building HTA capacity in Southeast Asia. Networks form an important element of connecting researchers with one-another and institutional arrangements to attract and retain staff trained in HTA is another area that needs to be considered [24]. Online resources such as the Guide to Health Economic Analysis and Research (GEAR) also offer researchers in LMICs to connect with global experts in the field [25]. In the pandemic era, online trainings and courses have made availability of technical trainings more accessible.

Category 3 reasons suggest an ideological resistance to HTA and the use of metrics such as cost per quality-adjusted life year saved to guide research allocation decisions. Interestingly, the reason of HTA placing a “price on life” was more frequently raised by respondents from upper middle and high-income countries, suggesting that the ideological resistance to HTA is not limited to low or lower-middle income countries. Opportunity costs are a fundamental reality of decision-making when resources are limited, and therefore willingness-to-pay thresholds or a “price on life” are simply an explicit and quantitative acknowledgement of that reality. As healthcare budgets are constrained worldwide, it is incumbent on a range of stakeholders (health professionals, patients, the media, donors and funders) to provide pressure on policymakers to make evidence-informed decisions. Communicating the importance of HTA, and concepts such as opportunity costs, will be critical in demonstrating that HTA is not only a technical exercise but also a transparent and inclusive process that can accommodate the values and principles that stakeholders affected by priority-setting decisions hold dear.

Furthermore, use of HTA is aligned with the UHC agenda as part of the Sustainable Development Goals (SDGs), which all governments have subscribed to [26]. Sustainability of any UHC programme relies on prioritising healthcare resources and recognising the opportunity costs in providing health services. As the results show, many respondents believe that HTA research would be ignored by policymakers which is why having a strong mandate for using HTA research in decision-making in LMICs, potentially in the context of UHC is imperative. The overwhelming response from this survey suggests that there remains limited understanding of HTA amongst users. This can be alleviated through trainings of potential users and sharing experiences with countries that have successfully applied HTA. This does not come without a cost and governments will need to invest in establishing HTA agencies and systems, which is well worth the money, as demonstrated by a study on the value of HTA agencies [1].

There have been several studies that have sought to understand the barriers to uptake of HTA in countries. These include lack of technical capacity, the limited knowledge of HTA among potential users and the need for better approaches for involving stakeholders [27, 28]. A review of HTA agencies in Asia showed that characteristics of the decision-making process for health, such as silo-based decision-making, poor quality of criteria for decision and the environment for conducting and disseminating research, could impede the use of HTA [29]. A study on prioritising the technical and context-specific issues for HTA shows that there is a dearth of quality data to conduct the analysis and limited integration of economic evaluations in decision-making [20]. The reasons expressed in the survey align well these common barriers but also reflect some of the additional hurdles that must be overcome by users.

There are limitations to this survey. It was administered online and through existing networks of the authors. There may therefore be hidden selection bias. The survey was intended to be short, simple and easy to complete to maximise the number of respondents and therefore does not include more details on the barriers encountered in HTA. This could be built on in future studies. Furthermore, to minimise the burden to respondents and to aid the analysis of results, participants were required to select their responses for two of the questions from a pre-defined list which was developed following a pilot, rather than enter free-form responses. There was also no option in the survey to decline all of the listed reasons. As stated in the results, most respondents felt that the list of pre-defined responses was comprehensive. The survey was advertised to a broad audience through social media channels and advertisement on institutional websites, it was therefore not possible to determine the total number of prospective participants. Hence, we were unable to calculate the response rate, nor are we able to comment on the characteristics of non-respondents. That said, it may be noted that there was higher representation of countries in Asia compared to other regions, as a result of the survey being launched at PMAC and the demographics of HITAP’s network. Future research might build upon this work by linking the reasons for not using HTA to the levels of HTA institutionalisation in different countries and the functions or mandates of HTA bodies, in countries where they are present. However, at present there is no established classification system for stages of HTA institutionalisation within countries, so this type of analysis was not undertaken. Future research could also use the responses in this survey to scrutinise the validity of the responses at a country level. It would also be beneficial to repeat surveys of this type over time to establish how reasons for not using HTA are changing over time.

The results of this study show that there is still more for local and international organisations to do to improve the understanding of HTA, potentially through training, educational activities and stakeholder engagement. HTA practitioners also need to consider how issues such as equity and affordability can be better incorporated into HTA methods to allay the concerns of those opposed to HTA. Though methods for the incorporation of equity into HTA have been developed, they remain underutilised [19, 30]. Methods of communication with policymakers, researchers and civil society also need to be improved on these issues. Despite capacity building efforts in LMICs, the responses suggest that capacity issues remain a widespread issue. This may be due to insufficient technical capacity, which could be due to a lack of institutional investment to maintain technical skills among staff or limit staff turnover when technical capacity has been built. It could also suggest that the capacity building efforts have been insufficient or are not reaching everyone. However, there have been efforts to modify the methods of HTA to better match capacity constraints in LMICs [31]. Despite the reservations that some have towards HTA, it remains a vital tool for improving health outcomes and it is important that we don’t make the “perfect the enemy of the good”. Given the results of this survey, we strongly believe that there is no justifiable reason for not using HTA to inform health care decisions: we have the tools and need to take the next step in implementing HTA to ensure that we allocate resources to their best possible use and improve healthcare for the populations of countries around the world.

## Supplementary Information

**Additional file 1: Questionnaire.** Note: The original survey used the term ‘excuse’ instead of reason. The list of reasons in the analysis includes one additional reason that was included in the first iteration of the survey “Requires competence and capacity that is scarce and needed for other purposes”.

**Additional file 2: Table S1.** Summary table of respondent characteristics and results.

## Data Availability

All relevant data are available in the supplementary materials.
