# Supplementary material for: Avoiding health technology assessment: a global survey of reasons for not using health technology assessment in decision making
Source: Cost Eff Resour Alloc. 2021 Sep 22;19:62. doi: 10.1186/s12962-021-00308-1 (PMC8456560; doi:10.1186/s12962-021-00308-1)
Supplement: Supplementary file 2 — Additional file 2: Table S1. Summary table of respondent characteristics and results. [file 12962_2021_308_MOESM2_ESM.pdf]

Top Excuses for Not Using CEA (Cost-Effectiveness Analysis)

* Required

1. Email address *

2. By proceeding, you agree for your results to be analysed and disclosed anonymously *

Mark only one oval.

- I got it!

Personal information

Please provide you personal informatio

3. Full name (first name - last name) *

4. Affiliation *

5. Primary country you work in *

Mark only one oval.

- Afghanistan
- Akrotiri
- Albania

- Algeria
- American Samoa
- Andorra
- Angola
- Anguilla
- Antarctica
- Antigua and Barbuda
- Argentina
- Armenia
- Aruba
- Ashmore and Cartier Islands
- Australia
- Austria
- Azerbaijan
- Bahamas, The
- Bahrain
- Bangladesh
- Barbados
- Bassas da India
- Belarus
- Belgium
- Belize
- Benin
- Bermuda
- Bhutan
- Bolivia
- Bosnia and Herzegovina
- Botswana
- Bouvet Island
- Brazil
- British Indian Ocean Territory
- British Virgin Islands

- Brunei
- Bulgaria
- Burkina Faso
- Burma
- Burundi
- Cambodia
- Cameroon
- Canada
- Cape Verde
- Cayman Islands
- Central African Republic
- Chad
- Chile
- China
- Christmas Island
- Clipperton Island
- Cocos (Keeling) Islands
- Colombia
- Comoros
- Congo, Democratic Republic of the
- Congo, Republic of the
- Cook Islands
- Coral Sea Islands
- Costa Rica
- Cote d'Ivoire
- Croatia
- Cuba
- Cyprus
- Czech Republic
- Denmark
- Dhekelia
- Djibouti

- Dominica
- Dominican Republic
- Ecuador
- Egypt
- El Salvador
- Equatorial Guinea
- Eritrea
- Estonia
- Ethiopia
- Europa Island
- Falkland Islands (Islas Malvinas)
- Faroe Islands
- Fiji
- Finland
- France
- French Guiana
- French Polynesia
- French Southern and Antarctic Lands
- Gabon
- Gambia, The
- Gaza Strip
- Georgia
- Germany
- Ghana
- Gibraltar
- Glorioso Islands
- Greece
- Greenland
- Grenada
- Guadeloupe
- Guam
- Guatemala

- Guernsey
- Guinea
- Guinea-Bissau
- Guyana
- Haiti
- Heard Island and McDonald Islands
- Holy See (Vatican City)
- Honduras
- Hong Kong
- Hungary
- Iceland
- India
- Indonesia
- Iran
- Iraq
- Ireland
- Isle of Man
- Israel
- Italy
- Jamaica
- Jan Mayen
- Japan
- Jersey
- Jordan
- Juan de Nova Island
- Kazakhstan
- Kenya
- Kiribati
- Korea, North
- Korea, South
- Kuwait
- Kyrgyzstan

- Laos
- Latvia
- Lebanon
- Lesotho
- Liberia
- Libya
- Liechtenstein
- Lithuania
- Luxembourg
- Macau
- Macedonia
- Madagascar
- Malawi
- Malaysia
- Maldives
- Mali
- Malta
- Marshall Islands
- Martinique
- Mauritania
- Mauritius
- Mayotte
- Mexico
- Micronesia, Federated States of
- Moldova
- Monaco
- Mongolia
- Montserrat
- Morocco
- Mozambique
- Namibia
- Nauru

- Navassa Island
- Nepal
- Netherlands
- Netherlands Antilles
- New Caledonia
- New Zealand
- Nicaragua
- Niger
- Nigeria
- Niue
- Norfolk Island
- Northern Mariana Islands
- Norway
- Oman
- Pakistan
- Palau
- Panama
- Papua New Guinea
- Paracel Islands
- Paraguay
- Peru
- Philippines
- Pitcairn Islands
- Poland
- Portugal
- Puerto Rico
- Qatar
- Reunion
- Romania
- Russia
- Rwanda
- Saint Helena

- Saint Kitts and Nevis
- Saint Lucia
- Saint Pierre and Miquelon
- Saint Vincent and the Grenadines
- Samoa
- San Marino
- Sao Tome and Principe
- Saudi Arabia
- Senegal
- Serbia and Montenegro
- Seychelles
- Sierra Leone
- Singapore
- Slovakia
- Slovenia
- Solomon Islands
- Somalia
- South Africa
- South Georgia and the South Sandwich Islands
- Spain
- Spratly Islands
- Sri Lanka
- Sudan
- Suriname
- Svalbard
- Swaziland
- Sweden
- Switzerland
- Syria
- Taiwan
- Tajikistan
- Tanzania

- Thailand
- Timor-Leste
- Togo
- Tokelau
- Tonga
- Trinidad and Tobago
- Tromelin Island
- Tunisia
- Turkey
- Turkmenistan
- Turks and Caicos Islands
- Tuvalu
- Uganda
- Ukraine
- United Arab Emirates
- United Kingdom
- United States
- Uruguay
- Uzbekistan
- Vanuatu
- Venezuela
- Vietnam
- Virgin Islands
- Wake Island
- Wallis and Futuna
- West Bank
- Western Sahara
- Yemen
- Zambia
- Zimbabwe

Excuses for not using HTA

Please use the listed excuses to answer question 1-3. You may propose additional excuses you have encountered in your setting that have not been included in the list in question 4.

1. It puts a price on life and rejects the intrinsic value of lives and health
2. It discriminates (e.g. the poor, old, disabled, rural populations), ignores distributional impact and justifies leaving populations behind
3. It neglects other valued outcomes beyond health
4. It ignores or hinders innovation by undermining rewards for R&D
5. It neglects political feasibility, e.g. the health care demands of powerful constituencies
6. It is all about cost control and cost cutting
7. It promotes privatisation and two-tiered systems
8. It ignores the need for deliberation and a participatory process
9. It is a technocratic, time consuming, and data hungry exercise
10. It relies on dubious estimates of costs and benefits, especially for certain interventions.
11. No local data as CEA requires competence and capacity that is scarce and needed for other purposes

12. It is irrelevant for countries with very limited budgets
(e.g. cost-effectiveness information is unlikely to further improve efficiency)

13. It is unnecessary as we are already prioritising highly
cost-effective interventions

14. It denies that we know enough to act and should not spend
resources on ever more evaluations

15. No acceptable decision rules for CEA (e.g. cost-effectiveness
threshold) to be used explicitly

6. 1. What is the excuse you find most often for not using HTA in the setting you work in? *

Mark only one oval.

- 1. It puts a price on life and rejects the intrinsic value of lives and health
- 2. It discriminates (e.g. the poor, old, disabled, rural populations), ignores distributional impact and justifies leaving populations behind
- 3. It neglects other valued outcomes beyond health
- 4. It ignores or hinders innovation by undermining rewards for R&D
- 5. It neglects political feasibility, e.g. the health care demands of powerful constituencies
- 6. It is all about cost control and cost cutting
- 7. It promotes privatisation and two-tiered systems
- 8. It ignores the need for deliberation and a participatory process
- 9. It is a technocratic, time consuming, and data hungry exercise
- 10. It relies on dubious estimates of costs and benefits, especially for certain interventions.
- 11. No local data as CEA requires competence and capacity that is scarce and needed for other purposes
- 12. It is irrelevant for countries with very limited budgets (e.g. cost-effectiveness information is unlikely to further improve efficiency)
- 13. It is unnecessary as we are already prioritising highly cost-effective interventions
- 14. It denies that we know enough to act and should not spend resources on ever more evaluations
- 15. No acceptable decision rules for CEA (e.g. cost-effectiveness threshold) to be used explicitly

7. 2. What is the excuse that you most strongly disagree with? *

Mark only one oval.

- 1. It puts a price on life and rejects the intrinsic value of lives and health
- 2. It discriminates (e.g. the poor, old, disabled, rural populations), ignores distributional impact and justifies leaving populations behind
- 3. It neglects other valued outcomes beyond health
- 4. It ignores or hinders innovation by undermining rewards for R&D
- 5. It neglects political feasibility, e.g. the health care demands of powerful constituencies
- 6. It is all about cost control and cost cutting
- 7. It promotes privatization and two-tiered systems
- 8. It ignores the need for deliberation and a participatory process
- 9. It is a technocratic, time consuming, and data hungry exercise
- 10. It relies on dubious estimates of costs and benefits, especially for certain interventions.
- 11. No local data as CEA requires competence and capacity that is scarce and needed for other purposes
- 12. It is irrelevant for countries with very limited budgets (e.g. cost-effectiveness information is unlikely to further improve efficiency)
- 13. It is unnecessary as we are already prioritising highly cost-effective interventions
- 14. It denies that we know enough to act and should not spend resources on ever more evaluations
- 15. No acceptable decision rules for CEA (e.g. cost-effectiveness threshold) to be used explicitly

8. 3. Why do you most strongly disagree with the excuse you selected in Question 2? *

Top Excuses for Not Using CEA (Cost-Effectiveness Analysis) 23/9/2563 BE 13:37

9. 4. Please propose the excuse that you encounter in the setting you work which have not ye been included in the list provided. *

Subscribe to this project, HITAP and iDSI updates

10. Would you like to subscribe for e-mail updates about this project? *

Mark only one oval.

- Yes
- No

11. Would you like to subscribe for e-mail updates about HITAP and iDSI *

Mark only one oval.

- Yes
- No

12. How do you know about this survey? *

Mark only one oval.

- Twitter
- Facebook
- LinkedIn
- HTA/HEOR networks I am a part of
- E-mail from HITAP's Communication Unit
- E-mail from colleagues/friends

This content is neither created nor endorsed by Google.

Google Forms

Top Excuses for Not Using CEA (Cost-Effectiveness Analysis) 23/9/2563 BE 13:37
